# Foreign Hospital Reports

**Published:** 1839-12-14

**Authors:** W. P. Johnston

**Affiliations:** Georgia


### MEDICAL EXAMINER.

DEVOTED TO MEDICINE, SURGERY, AND THE COLLATERAL SCIENCES.

No. 50.] PHILADELPHIA, SATURDAY, DECEMBER 14, 1839. [Vol. II.

### FOREIGN HOSPITAL REPORTS.

[By W. P. Johnston, M. D., of Georgia.)

### HÔTEL DIEU—SERVICE OF M. LOUIS.

*Case of Cancer of the Stomach—Absence of lancinating pains—Tumour felt in the region of the small curvature—Great distention, with evident contraction of the stomach, perceptible to the eye—Death—At the autopsy, scirrhus of the pylorus and small curvature—General hypertrophy and dilatation of the stomach—Mucous membrane generally healthy, &c. &c.—Remarks.*

M., sixty years of age, a soldier during seven years, since a merchant of hardware in the streets of Paris; he is of ordinary height, moderately thin, and possesses sufficient intelligence; his habits are rather intemperate; his diet has generally been sufficiently good and substantial; health ordinarily very good; says he had a fever at the age of seventeen, which lasted one or two months. In the month of August, 1838, this patient commenced to vomit occasionally. Previous to this it does not appear that there had been any abnormal symptom in the digestive apparatus; his appetite had been good; he had never experienced eructation of gas from the stomach, nor any unpleasant sensation in the region of this organ; digestion had appeared to be perfect. The vomitings, once commenced, became gradually more and more frequent; first, at intervals of several days, and finally, of late, as often as several times a day; sometimes they occurred immediately after a repast, but more generally at the end of several hours. The matter ejected was of a sour taste, and in it the patient often remarked substances which he had eaten four or five days before; the vomitings were never of a black or brownish colour. The acid eructations commenced about the same time with the vomitings, and soon became of frequent occurrence; he has not experienced any pain in the stomach, nor even a sensation of weight; the appetite has gradually diminished; and the emaciation, in the opinion of the patient, has become considerable, though he was never remarkable for embonpoint. The stools became less frequent, and finally rare,—but one during the last eighteen days.

Up to the present time the patient has continued his fatiguing trade, which has obliged him to walk more or less from morning to night; twice, however, he has been obliged to enter the hospital, and to remain a few weeks; while there the vomitings became rather less frequent.

*Actual state, the 26th February*, 1839, *two days after entrance.*—The character of the patient seems rather gay; intelligence and senses untouched; expression natural; face moderately coloured; emaciation to the second degree; tongue moist, whitish posteriorly; appetite variable; thirst moderate; sour eructations; vomiting yesterday evening composed, in part, of prunes, which the patient eat twenty-four hours before; no pain nor abnormal sensation at the stomach; two stools after injection given yesterday; abdomen not developed; cicatrices of cauteries in the epigastric region; the left flank is less depressed than the right; considerable depression below the xyphoid cartilage, a little above the umbilicus; slight elevation. On palpating here we discover a tumour, irregular in surface, which appears to extend in a line running from left to right, to the same distance on each side of the median line of the body, but its limits cannot be well defined; pressure occasions little or no pain; percussion and auscultation of the heart and lungs reveal the normal physical signs; pulse sixty-two, small, regular; temperature of the skin natural. (℞. One bottle of water of Vichy; soup twice; injection.)

*Feb. 29th.*—Vomiting every day since last date,—generally in the evening, two or three hours after dinner; the matter ejected yesterday resembles half-digested bread; there is no appearance of the prunes eaten three hours before. The abdomen exhibits a development extending from the umbilicus to the distance of three inches above, and of an equal extent in breadth; the percussion here gives a clear sound, much more so than elsewhere; the tumour noticed on the 26th cannot be felt; no change in the other symptoms. (℞. Water of Vichy; gum potion; soup twice.)

*March 4th.*—The stomach is much distended by gas; it forms from its development a tumour in the epigastric region about three inches in extent, and of an oval shape, but varying every instant in size and form, owing to the contractions of the stomach, which are constant, and which occasion a kind of undulating motion, perceptible to the eye at a considerable distance from the bed. Vomiting daily, usually in the evening; appetite at times; pulse sixty. (℞. Water of Vichy; soup; chicken.)

*March 11th.*—The development and contractions of the stomach have been remarked daily; the distention now exists to a very slight dgree, and it is easy to satisfy one's self of the existence of a tumour; matter vomited yesterday of a grayish colour. (℞. Two bottles of water of Vichy; gum potion, &c.)

18*th.*—No sensible change since last date, except that the patient is becoming more feeble: the stomach, when distended, appears to occupy a larger space than usual. The matter vomited yesterday is of the consistence of mucus, adhering in part to the bottom of the vessel, and presents, for the first time, a brownish colour. The face exhibits an expression of disgust, and there is nausea; tongue moist, nearly natural; stools not more than one daily; pulse ninety-six; skin rather warmer than natural.

20*th.*—Vomiting of a bitter and reddish matter; the epigastrium being more depressed than usual, the tumour can be felt; it appears to M. Louis to be situated in the small curvature of the stomach; emaciation progressive. (℞. Same.)

21*st.*—Abdomen contracted, slight projection near the umbilicus; sensation of tumour; no vomiting since yesterday; no diarrhœa; pulse ninety-six, very small, difficult to count; sounds of heart not easily heard; extremities still warm.

22*d.*—Vomiting two or three times of a brownish and pultaceous substance; pulse ninety-six, very feeble; skin of natural temperature. (Same prescription.)

23*d.*—Brownish ejections from the stomach; gradual sinking; death between 8 and 9, P. M. Lungs not examined during the last few days, in consequence of the great feebleness of the patient; there had been no cough.

*Autopsy thirty-six hours after death.*—Atmosphere dry; last degree of marasmus; no stiffness of the extremities; walls of the abdomen a little livid. *Brain,*—moderate degree of infiltration under the arachnoid; the membranes can be easily removed without injuring the substance of the brain; they present their normal appearance. The cortical and medullary portions present nothing remarkable; a teaspoonful of limpid fluid in each lateral ventricle. *Chest—Heart,*—two or three spoonsful of a citron-coloured fluid in the pericardium; *heart,* three inches at its base, three inches and two lines from base to apex; the right ventricle contains a soft, black coagulum; none in left; the valves present their usual colour and suppleness; lining membrane of the several cavities exhibits nothing remarkable. The aorta presents near the semilunar valves some small white spots, situated under the lining membrane. *Lungs.*—No adhesions; the posterior and inferior portion of each lung is brownish red, slightly granulated, and a little friable. Bronchial tubes apparently healthy. *Abdomen.—Stomach* very much distended and enlarged; pushes up the left lung, and is partly concealed by the border of the liver; it contains a considerable quantity of a brownish liquid, as well as gas. The œsophagus is much dilated between the seventh vertebra and the cardiac orifice; incising it, we find it measuring two and a half inches near this latter point,—in thickness it measures from one to one and a half lines; this is principally owing to the great hypertrophy of the muscular coat; the internal surface is slightly grayish, and presents near the cardiac orifice of the stomach several irregular whitish points, (plaques,) which seem to be the remains of the epithelium. *Stomach exteriorly.*—On its anterior surface we remark an injection of some of the larger venous trunks. On the posterior surface, and near the pylorus, is a vegetation, (plaque,) more or less circular, five lines in diameter, elevated above the surrounding parts, of an irregular, mammelonnated surface, and of a yellowish colour; this point is excessively hard, grating under the knife; around is a slight depression, with lines or radii running from it, resembling certain cicatrices. *Interiorly,* we find a portion of the small curvature near the pylorus occupied by a tumour two inches and three lines long, and one inch and a half in height; its greatest thickness, taken half an inch from the pylorus, is ten lines. On the posterior part, and on the side near the œsophagus, the tumour stops abruptly. On the anterior surface, on the contrary, it diminishes gradually, confounding itself insensibly with the general increased thickness of the coats of the stomach. Towards the pylorus the tumour also ceases abruptly; about one-third of this orifice is invaded by it, and this has occasioned so great a diminution in its diameter that it was impossible to introduce the extremity of the little finger before we had opened it. Near the pylorus is a depression in extent about an inch square; greatest depth one line; smooth; of of a bluish colour. Here the mucous membrane is entirely wanting, and the cellular tissue forms the base of the ulcer, except near the centre, where the muscular tissue is exposed. When we cut into and carefully examine this tumour, we find that it is composed, 1st, of the submucous cellular tissue very much hypertrophied, offering from two to three lines in thickness near the pyloric valve, of a whitish colour, indurated, grating under the scalpel, in other words, converted into scirrhous matter; 2d, of the muscular coat also greatly hypertrophied, four lines in certain points, and easily recognizable by its transparent and rosy appearance. The cellular tissue between the muscular and peritoneal coats does not appear to have undergone any alteration, nor to be thicker than usual. In certain points of the tumour, viz., where it offers its greatest thickness, it is impossible to recognize the different tissues of the stomach. The walls of the stomach generally are much thicker than usual, viz. three-quarters of an inch in the anterior surface, and still more so near the large curvature; to the touch the stomach is less flexible than usual. The mucous membrane is of a grayish colour, and appears to have its normal thickness and consistence, except near the ulceration noticed, where it is thickened and softened in the extent of about four lines; it presents an injection of some of the larger trunks on both the anterior and posterior surface. The *duodenum* and the small intestines contain a brownish liquid, similar to that found in the stomach, but less abundant. The mucous membrane offers its usual physical characters. The large intestine is filled with firm, fecal matter; the mucous membrane can be raised in strips of nearly an inch long; thickness normal. *Mesenteric glands.*—Some are of the size of a French bean, (haricot,) of a grayish or rosy colour internally; there are other glands much smaller, whitish internally, and indurated, resembling scirrhous matter. *Liver.*—Usual size, reddish colour, good consistence; gall bladder distended by a thick and dark-coloured bile; spleen and kidneys offer nothing remarkable.

*Remarks.*—Much difference of opinion prevails as to the cause of cancer, especially of the stomach. M. Andral classifies the disease among the chronic inflammations of the stomach, and believes it to be constantly preceded by a chronic gastritis, even in those cases where, at the autopsy, the mucous membrane has been found healthy. Broussais, Bouillaud, and others, entertain a similar view, while Louis from observation and analogy, has been led to deny this relation of cause and effect. Gastritis, according to this latter observer, is very rare, since during five years while at "La Pitié," he saw but five cases. Again, he has always found inflammation of the stomach less marked in the region of the pylorus, which is, on the contrary, the chosen seat for cancer, since he has found it here as often as in all the other parts of the stomach put together. 3dly. M. L. has never found cicatrices except in the posterior face of the stomach, the point where cancer is the most rare. 4thly. Inflammation of the lungs is very frequent, while cancer of these organs is very rare; inflammation of the liver, on the contrary, is rare in this climate, while cancer of this organ is not unfrequently met with. It is probable that both parties are too exclusive on this point. Admit with the majority of authors, that there exists among certain individuals, a predisposition to this disease, or a cancerous diathesis, and we can then easily conceive how an inflammation of the stomach may become the accidental cause of the development of scirrhus in the stomach, in the same manner as a blow upon the breast appears, in some instances, to have been the cause of scirrhus of the mammary gland; or as some local irritation, like that occasioned by continual smoking of a pipe, becomes, evidently, in certain cases, the cause of the development of mucous tubercles near the commissures of the lips in persons whose constitutions are labouring under a syphilitic influence.

In the case which we have detailed above, it appears probable that the disease remained completely latent for a time, and that it was not until the pyloric orifice was so far diminished in size as to interfere with the healthy functions of the stomach, that the vomitings, the first appreciable symptom, commenced.

In the earlier stages of scirrhus of the stomach, much difficulty is experienced in making a correct diagnosis, in consequence of the great analogy of its symptoms with those of chronic gastritis. M. Andral, in fact, declares that except in those cases where a tumour can be felt, there is no certain sign to distinguish the one affection from the other. The acid eructations, the vomiting of a brownish matter like coffee grounds, the lancinating pains, &c., which are regarded as belonging more especially to cancer, have all been seen by him in cases where the autopsy has revealed nothing but a chronic gastritis, while, on the other hand, one or more of these symptoms are often wanting in genuine cancer—as the above case presents an example of, with regard to the absence of lancinating and other pains. When the the disease becomes a little more advanced, especially if it occupies one of the orifices, we have soon a series of phenomena, which enable us in the majority of cases, to diagnosticate not only the disease, but its particular seat. The discovery of the tumour is still, undoubtedly, the most important sign of this disease, as M. Andral has remarked, and a careful palpation should always be practised. If a tumour be once felt, there exists no difficulty in deciding to what organ it belongs. If it be the liver or the spleen, it is generally easy to recognise the sharp and regularly defined edge of these organs: if it be in the integuments, it will not change its position, when the patient is placed upon the side: if it be in the stomach, by pressing on it, and making the patient take a full breath, it will be found to rise and fall with the diaphragm, and if the patient places himself upon the side, the tumour will be found to have changed position also. Making the patient change his position is very important, for it sometimes happens, when the disease is seated near the pylorus, that the tumour cannot be felt when the patient lies on his back, but if we place him on the left side, the tumour is thrown more forward and we can then detect it.

The dilatation and distention of the stomach by gas, which accompany scirrhus with contraction of the pylorus, are sometimes sufficient to enable us to judge of the form and size of this organ through the walls of the abdomen. It is rare, however, that we have an occasion of seeing the contractions so satisfactorily as in the above case.

—

LA CHARITÉ—SERVICE OF M. VELPEAU.

*Case of Varix of the Lower Extremities—Operation—Death.*

Peter Galopan, aged 34 years, a white leather dresser by profession, possessed of a good constitution, but of a nervous and irritable temperament, entered the hospital "La Charité," the 1st March, 1839, Salle Ste. Vierge, service of M. Velpeau. The profession of this man has obliged him to remain standing almost constantly; no other cause can be assigned for the existing disease. The enlargement of the veins appears to have commenced in the right leg, but for several years since they have both been affected; the varicose tumours have progressively augmented in size, although the patient has made habitual use of a compressive bandage.

*Actual state.*—The left leg offers a series of tumours, soft and elastic, with a bluish colour of the integuments in their most prominent points. These extend on the internal surface of the leg, from the upper part of the thigh, as far as the internal malleolus, marking out the passage of the internal saphena vein. Varicose veins less voluminous are seen upon the upper surface of the foot and the external part of the leg. On the internal side, and near the instep, is a small ulceration which appears to be undergoing the process of cicatrization; it is surrounded by reddish coloured cicatrices evidently of new formation. The integuments offer a considerable number of vascular arborisations of a violet colour, formed by the dilated capillary vessels.

On the right side, the varicose veins are fewer in number, and their volume is much less considerable. The patient says that he has remarked at times, in the evening, a little swelling about the ankles.

*April 4th.*—Nothing new has occurred in the state of the patient. His general health continues perfectly good. Mr. Velpeau passed 12 pins under the varicose veins of the left leg, and passing a strong ligature under each pin, tied them as tightly as possible, so as to stop entirely the circulation in that part of the integuments which was thus included. This operation produced an acute pain of short duration.

During the day, the patient experienced some slight pain. In the evening we remarked the commencement of phlyctena accompanied by a slight redness upon several of the points which were strangulated.

*April 5th.*—No general reaction: the pain from the ligatures has almost entirely ceased.

From the 6th to the 10th. The skin acquires a brownish colour in the points strangulated by the ligatures, and becomes insensible; around these is a circular redness, perfectly circumscribed; the process of elimination between the dead and the living portion seems to have commenced.

The varicose veins have diminished considerably in size, and the circulation appears to be interrupted; however, there are one or two tumours in which the blood appears to have gained access by the collateral branches.

M. Velpeau applied two new ligatures in the same manner as before. Five or six days after the operation, the ligatures were cut and the pins withdrawn; one of those near the knee, had become bent during the application of the ligature, and its extraction was both difficult and painful.

*April 16th.*—Last night the patient was seized with intermitting chills, which lasted until near morning. He vomited several times a matter of greenish colour. This morning, expression of exhaustion; the nausea and vomiting continue; the tongue is furred; mouth clammy, with a bitter taste. A little headach: and at times, vertigo; great heat of the skin; pulse small and frequent. The leg is swollen; red lines are seen over its surface; the inguinal glands are swollen and painful to the touch. This "angioleucite" appears to have been occasioned by the ulcerations which succeeded the sloughing of the strangulated portions of the integuments; the red lines which appear to mark out the course of the lymphatic vessels, seem more numerous and more marked in the neighbourhood of the point where the removal of the pin was found so difficult, and which has not ceased to be more painful than the others.—℞. Venesection.

*April 17th.*—Delirium during the night: this morning the expression of the patient is still more unfavourable; a little stupor; prostration; general trembling; headach; the patient sees flashes of light; noise in the ears; contusive pain in the lumbar region and in the articulations. Tongue dry and incrusted; intense thirst; vomiting has ceased; the epigastrium is slightly painful on pressure; sensation of a liquid in the intestines; three liquid stools since yesterday. Skin hot; pulse small and frequent.

*April 18th.*—Delirium during the whole night, and continuing still. The symptoms observed yesterday, still exist, but much more marked; profound stupor; trembling of the extremities; subsultus tendinum; the face is of a violet colour; lips swollen and blackish; tongue considerably swollen, dry and livid; skin hot; pulse thread-like, and very frequent. Bluish spots exist upon several points of the body; they are more marked on the internal surface of the arms: upon the back of the right hand is a small fluctuating tumour.

*April 19.*—Delirium during the whole night; the stupor is extreme; the patient no longer answers the questions addressed to him. Face livid; lips swollen; pulse very frequent; scarcely sensible. The livid spots noticed yesterday over the body have augmented in intensity and in extent; the tumefaction on the back of the right hand is not so great, but it is increased in extent and offers a bluish colour; considerable swelling of the left leg; the extremities commence to be cold.

Death, at 9 A. M.

*Autopsy.*—Twenty-four hours after death. Atmosphere dry.

Lividity of the teguments; bluish lines mark the course of the subcutaneous veins; abdomen of a greenish colour.

*Thorax.*—The right lung is attached to the costal pleura throughout, by old adhesions. At the summit of the left lung exists a cretaceous tubercle. Passive engorgement of the two lungs in their posterior portion, from which a pretty considerable quantity of bloody serum can be pressed out. Nothing else remarkable.

*The heart* contains a quantity of blood, partly fluid, partly forming a clot, of but little consistence. A small, yellowish fibrinous coagulum in the right ventricle.

*Abdomen.*—Stomach and intestines distended by gas. A few inches above the ilio-cœcal valve a patch (plaque) of Peyer's glands, red, swollen, and slightly ulcerated; the follicles are prominent.

Through the incisions made in the points of the integuments, which presented a bluish colour, we remark an effusion of blood, varying in quantity, in the subcutaneous cellular tissue; the greater part of this blood is still fluid, and escapes through the incisions, but there exists some clots of slight consistence. The effusion of blood, which exists on the abdomen, legs, and especially on the inner surface of the arms, penetrates in the intervals of the muscles and fills the sheath of the vessels. The tumefaction observed on the back of the hand is owing also to an exhalation of blood. The inferior vena cava is distended by black and fluid blood. In the saphena veins is also seen very fluid blood, with some few clots but little consistent. Near the superior part of the left thigh the saphena vein is surrounded by a mass of enlarged lymphatic ganglions: upon incising these, we remark a notable injection, which diminishes from the circumference to the centre. At this point, that is, near the junction of the saphena with the crural vein, we find the vein (saphena) obliterated, in the extent of about eighteen lines, by a coagulum adhering to its internal membrane, which is red, and presents an irregular surface. Corresponding to the pin placed nearest the lowest extremity of the thigh exists another coagulum, also adhering, about eight or nine lines long, of a bright red colour; at this point the vein is contracted in its calibre. Corresponding to the other ligatures there is neither coagulum nor adherence of the sides of the vein, but if we place the vein between the eye and the light, the points where the pins passed are marked by a transparent line, and it appears that there is evidently in these points a destruction of part of the middle tunic of the vein, the internal and outer coat remaining untouched. The passage of the pin through the cellular tissue is indicated by a canal, red, and lined by a false membrane. The saphena vein presents a notable thickening of its coats; on the inner surface, which is of a greenish white colour, we remark an areolar structure, which appears owing to the projection of the fibres of the middle coat; this is hypertrophied, and evidently formed of fibres, of which the greater part run in a longitudinal direction, and present, in certain points, a muscular aspect; on the internal surface we observe, also, certain thread-like prolongations, which are attached only at their two extremities; these, in some points, bear a strong resemblance to the columnæ carneæ of the right ventricle of the heart; corresponding to the points where the varicose tumours existed, before the operation, these bridles are placed, the one above the other, so as to give an erectile appearance to the internal surface of the vein. This latter disposition is especially remarkable in the portion of the vein corresponding to the inferior third of the thigh; here the vein appears to be divided into secondary cavities; here also we remark a lateral dilatation of the vein, the walls of which appear to be formed by the external and internal coats alone, while the middle appears to have entirely disappeared. In the vein of the opposite side we remark the same hypertrophy of the different coats, but to a less degree.

*Remarks.*—We pass over the peculiar points of interest which the above case presents, in a pathological point of view; we shall simply say a few words upon M. Velpeau's method of operating for varicose veins. This method is undoubtedly far more simple, and less dangerous than the ancient methods, especially that of cutting the veins; but the above case proves that it may sometimes be fatal. In his lecture on this subject, some time since, M. V. declared that up to that period, of upwards of one hundred patients, upon whom he had practised this method, he had never lost but this one case.

There are three objections which we think may reasonably be urged to this operation.

1st. The operation is rarely necessarily demanded, varicose veins being seldom more than an inconvenience.

2d. The operation does not fulfil the object proposed by the surgeon. The anastomosis between the deep seated and superficial veins of the lower extremity is so numerous that it becomes a difficult matter to obliterate a dilated vein, and when effected, even partially, others become dilated in their turn. We have often remarked after this operation only a moderate and partial diminution in the volume of the vein. A few days since we examined a patient, a considerable time after the operation; the internal saphena vein of the left leg was much dilated between the points where the ligatures had been applied, as indicated by the cicatrices. At times this method of treatment fails entirely. An example of this we had an occasion of seeing a few months since, in a medical student operated upon for varicocele, in the wards of M. Velpeau; he had been discharged as cured, but in three or four weeks returned again; on examination the veins were found more numerous, and more dilated than before the operation.

3d. Considering the above objections, we do not think that the imperfect cure which follows the operation a sufficient compensation for the danger to be incurred. The mortality, numerically speaking, has been very small, but in proportion to the utility gained, perhaps, much greater. It must be recollected that this operation is more generally performed in hospital practice, and here the danger must always be greater and the success more uncertain, in consequence of the frequent prevalence of an epidemic erysipelas. In private practice, it is true, this objection would not be so tenable.

A few days since, M. Breschet lost a patient, from phlebitis, after the application of his instrument for varicocele; this is the first serious accident that M. B. has remarked after his operation. It is very important to note here, that this patient, a young Englishman, was operated upon by Sir Astley Cooper, and his case has been published in one of the English Medical Journals as a radical cure; the disease, however, returned.

Within a few days, (Oct. 15) M. Velpeau has lost a second patient, after the operation for varix. He was attacked by erysipelas of the thigh and died with symptoms of purulent absorption. At the autopsy, pus was found in the veins, and in the cavity of the pleura. Another patient operated upon for varicocele, about the same period, was seized with erysipelas of the scrotum, accompanied by chills, high fever, and alteration of the natural colour of the skin, which became of a yellowish tinge. His condition has been extremely alarming, and the issue is still very doubtful. It must be remarked, however, that in these two patients, and in others operated upon recently, M. Velpeau has employed the method of M. Gagnebé, which consists in passing a ligature around the vein, under the integuments, by a simple puncture of the skin.

—

HÔTEL DIEU—SERVICE OF M. LOUIS.

*Case of Amaurosis, consequent on Vinous Excess.—Cure.*

(Abridged from a Case read before the Societé Médicale d'Observation.)

S—, unmarried; 24 years of age; a mason by profession; entered Hôtel Dieu, service of M. Louis, the 4th February, 1839. On the 13th of January last he left the hospital, having been confined there several weeks by an illness, probably, from the account he gives, a typhoid fever; during the fifteen days which have preceded his present disease he recovered his embonpoint and his strength, and resumed his trade. The eyesight of this patient has always been very good, he is not a myops, never had any disease of the eyes; but about a year since, after a fall in which he struck his head, his sight was troubled, but this passed off in a day without treatment. While a boy he was subject for several years to a pain in the head, of which he was sometimes promptly relieved by a spontaneous epistaxis, which frequently occurred at this period. For the last four or five years he has often experienced vertigo to a slight degree. On Sunday, the 27th January, while still enjoying perfect health, the patient drank three or four bottles of wine, without becoming intoxicated. The next day he was seized with a pain in the head, and with diarrhœa, which have persisted ever since. The third day the sight became troubled. There was swelling of the face, and nausea. Complete loss of appetite since the commencement of the prodromes. The introduction of a small quantity of aliment, daily, produced eructations and enlargement of the abdomen. No fever thus far; no appreciable alteration in the other functions. The disturbance in the vision continued increasing, so that on the day of his entrance the patient could not walk without being guided, nor could he distinguish any of his friends. Thus far, no treatment.

After the examination made as to other possible causes of the disease, it appears that the patient has received no blow upon the head, nor been exposed to brilliant light; he has committed no venereal excesses, &c. We may remark, that thus far the occasional alcoholic excesses of S. have never produced any alteration in the organ of vision.

*February 5th.—Actual state.* Hair, light colour; eyes, gray. No emaciation. Face swollen, coloured, without pain, a sensation of tension only. Eyelids natural. No injection of the eyes; pupils, one to two lines in diameter, regular, not contracting even at the approach of a candle, nothing remarkable behind them. At the distance of about a foot the patient distinguishes nothing; at times he remarks sparks, black spots, &c. floating before his eyes; no pain, nor even sensibility to the light of a candle placed at the distance of four inches. Pain in the head general; no vertigo; sense of hearing unaffected. Tongue a little whitish, moist; moderate thirst; no appetite. Abdomen developed, not painful; one stool. Auscultation and percussion denote nothing remarkable in the lungs nor in the heart. Pulse seventy-eight, regular; heat of the skin natural.

℞.—Rice water with gum syrup, acidulated with lemon juice, gum potion with hydrated chloride of morphia, gr. j,—strict diet.

*Feb.* 6.—The face appears rather less swollen; no alteration in the pupils; blindness nearly complete, more so than yesterday.

℞.—Injection; bleeding at the foot; suppress the potion.

*Feb.* 7.—Yesterday, before bleeding, the patient was seized with a violent pain in the head, accompanied by nausea. An hour after the bleeding, head was relieved, and the vision a little better. To-day the pupils contract nearly as much as in their natural healthy state, and the sight is sensibly improved. At the distance of two feet, the patient now distinguishes the light of a candle. Slight pain, like sand in the eyes; no injection of the conjunctiva; cephalalgia frontal; no nausea since the bleeding.

℞.—Fifteen leeches behind each ear; one bottle of Seidlitz water.

*8th.*—The patient distinguishes objects at a greater distance; he sees as if through a fog. The contraction of the pupils more perfect; they have not more than the diameter of half a line at the approach of a candle. The sparks and black spots, seen by the patient constantly, since entrance, are now much less numerous. The face is reduced to its natural size. One stool; abdomen presents its natural volume. Pulse 60.

℞.—Discontinue Seidlitz waters; two bowls of soup.

*9th.*—Headach has ceased; sight only troubled when the patient regards steadily an object, and then only he observes spots before his eyes.

℞.—Sol. sir. gum. The eighth of a portion.

*11th.*—Sight as perfect as in health; he can regard, fixedly an object without remarking any spots. Feels quite well.

℞.—The half a portion.

The patient was discharged the next day, perfectly cured.

*Remarks.*—The example of amaurosis, which we have here given, seems to belong to that division which M. Sichel calls "amblyopie congestive cérébrale," or partial amaurosis, arising from more or less congestion of the brain, and not from any lesion of the retina. The previous history of the patient, which renders it not impossible that he was accustomed to an occasional congestion of the brain, though no doubt very slight; and especially the happy effect produced by blood-letting, seem to strengthen this idea. Alcoholic liquors, though often cited by authors as a cause of amaurosis, are so, we are induced to think, rather rarely; at least, we recollect of seeing but one other patient in whom the relation between cause and effect was sufficiently well marked to be admitted without a doubt. The congestive amblyopy, in the acute form, is decidedly the most manageable form of this disease, whatever may have been the exciting cause; a well-regulated antiphlogistic treatment is that which we must chiefly depend upon, and this will sometimes effect a cure in a very short time, as we have an example in the above case.

There is another form of congestive amaurosis, more chronic, and much more frequent; this is generally met with in women about the critical age, or in both sexes, from the suppression of an accustomed hæmorrhoidal flux. It is in this form that Mr. Sichel often produces a favourable modification in the symptoms by restoring the suppressed discharges, and by antiphlogistics, sometimes long continued, in order to remove all tendency to congestion before attempting the application of local stimulants, of strychnine, &c., which afterwards are required. These latter remedies are often too soon resorted to, in consequence of persons being too much led astray by the name of amaurosis, with which there is so strong a tendency to connect the idea of a torpidity of the retina, or of a lesion beyond cure.

We cannot but mention, in passing a third form of this disease, which merits great attention. It is that which is produced from the incessant use of the eyes in the examination of minute objects. We have frequently had occasion to see this, at the consultations of M. Sichel, among seamstresses. Here the pupils retain their contractibility, but the power of vision becomes enfeebled, the patient sees sparks, spots, &c., and is unable, at times, to bear a bright light, &c. Here there seems to be more of a nervous irritation than a congestion, and hence the treatment becomes difficult, as a nervous temperament, with local congestion or irritation, must be combated against at the same moment.

